# Olaparib synergizes with arsenic trioxide by promoting apoptosis and ferroptosis in platinum-resistant ovarian cancer

**DOI:** 10.1038/s41419-022-05257-y

**Published:** 2022-09-27

**Authors:** Sangsang Tang, Yuanming Shen, Xinyi Wei, Zhangjin Shen, Weiguo Lu, Junfen Xu

**Affiliations:** 1 Women’s Reproductive Health Laboratory of Zhejiang Province, Women’s Hospital, Zhejiang University School of Medicine, 310006 Hangzhou, Zhejiang China; 2 Department of Gynecologic Oncology, Women’s Hospital, Zhejiang University School of Medicine, 310006 Hangzhou, Zhejiang China; 3 Cancer center, Zhejiang University, 310058 Hangzhou, Zhejiang China

**Keywords:** Cell death, Targeted therapies, Ovarian cancer

## Abstract

Poly (ADP-ribose) polymerase (PARP) inhibitors are efficacious in treating platinum-sensitive ovarian cancer (OC), but demonstrate limited efficiency in patients with platinum-resistant OC. Thus, further investigations into combined strategies that enhance the response to PARP inhibitors (PARPi) in platinum-resistant OC are required. The present study aimed to investigate the combined therapy of arsenic trioxide (ATO) with olaparib, a common PARPi, and determine how this synergistic cytotoxicity works in platinum-resistant OC cells. Functional assays demonstrated that the combined treatment of olaparib with ATO significantly suppressed cell proliferation and colony formation, and enhanced DNA damage as well as cell apoptosis in A2780-CIS and SKOV3-CIS cell lines. Results of the present study also demonstrated that a combination of olaparib with ATO increased lipid peroxidation and eventually triggered ferroptosis. Consistently, the combined treatment synergistically suppressed tumor growth in mice xenograft models. Mechanistically, ATO in combination with olaparib activated the AMPK *α* pathway and suppressed the expression levels of stearoyl-CoA desaturase 1 (SCD1). Collectively, results of the present study demonstrated that treatment with ATO enhanced the effects of olaparib in platinum-resistant OC.

## Introduction

Ovarian cancer (OC) often presents at an advanced stage and remains the most lethal gynecological malignancy [1–3]. According to the data from American Cancer Society, ~21,410 cases will be newly diagnosed with OC and 13,770 cases will die from it annually [3]. Cytoreductive surgery followed by paclitaxel and platinum-based chemotherapy remains the standard therapy for newly diagnosed OC [2, 4, 5]. According to the disease-free interval (DFS), patients were categorized as platinum-sensitive or platinum-resistant with a cutoff of 6 months [6]. Despite the standard treatment options, an estimated 70–85% of patients with advanced OC would relapse within 2 years [7]. Many patients will often receive multiple lines of treatment following relapse, and each subsequent line of therapy is characterized by shorter DFS, and the cancer in almost all patients will develop into the platinum-resistance subtype [8]. Previous studies described multiple mechanisms underlying the resistance to platinum, including decreased accumulation of intracellular drug, enhanced ability of repairing DNA damage, intracellular inactivation of the drug and the inactivation of apoptotic programs [9, 10]. Despite of the complex mechanisms, platinum resistance is critical for the high mortality of OC.

Poly (ADP-ribose) polymerases (PARP) play a critical role in repairing DNA single-strand breaks (SSBs) [11, 12]. PARP inhibitors (PARPi) increase replication stress and genomic instability, leading to the accumulation of SSBs and subsequent DNA double-strand breaks (DSBs), thereby resulting in cell death [13, 14]. Several previous randomized controlled trails have demonstrated a notable improvement in progression-free survival (PFS) following the maintenance treatment of PARPi in patients with platinum-sensitive recurrent OC [15–17]. At present, platinum sensitivity is recognized as a functional indicator for PARPi application. It is estimated that >50% patients with platinum-sensitive OC benefit from PARPi monotherapy, but limited efficacy of PARPi monotherapy has been observed in patients with the platinum-resistant subtype (5–30%) [18–20]. Therefore, novel combination strategies are required to enhance PARPi-induced DNA DSBs. In order to overcome the inefficiency of apoptosis-inducing agents in platinum-resistant OC cells, further investigations are required to expand the efficiency of PARPi in platinum-resistant recurrent OC. Moreover, the role and pathogenesis of PARPi in platinum-resistant patients are yet to be fully characterized.

Ferroptosis, distinct from apoptosis, necrosis, and autophagy, was identified as a novel form of programmed cell death in 2012, which is characterized by the accumulation of iron-dependent lipid peroxides and the subsequent depletion of polyunsaturated fatty acid (PUFA) phospholipids [21]. Previous evidence has demonstrated that chemo-resistant cancer cells exhibit a specific sensitivity to lipid peroxidation [22–24]. Thus, triggering ferroptosis may be a potential method to overcome chemotherapy resistance in cancer cells. Notably, olaparib was demonstrated to induce ferroptosis, and synergized with ferroptosis inducers by repressing cysteine/glutamate transporter (SLC7A11) in breast cancer type 1 susceptibility protein (BRCA)-proficient OC cells [25].

Arsenic trioxide (ATO) is a Traditional Chinese Medicine used for the treatment of acute promyelocytic leukemia that has been approved by the US Food and Drug Administration for >20 years. In recent decades, previous studies have demonstrated the in vitro effect of ATO in numerous solid tumors by activating oxidative stress, enhancing DNA damage and ultimately inducing apoptosis [26–29]. Nakamura’s study revealed that ATO suppressed osteosarcoma growth via increasing the accumulation of DNA damage and promoting cell apoptosis [30]. However, due to its rapid renal clearance, the application of ATO in solid tumors requires a high dosage. Arsenic is a metalloid, the high dosage of its inorganic form (ATO) may cause severe toxicity, including cardiac failure and hepatosis. Therefore, further investigations into potential combination strategies are required, to reduce the dosage required and enhance the efficiency of ATO for clinical application. Results of previous studies demonstrated that arsenic compounds induced pancreatic dysfunction, testicular cell death and neuronal cell death by promoting ferroptosis through different mechanisms [31–34]. Meng’s study demonstrated that arsenate exposure induced ferroptosis in testis by triggering oxidative stress [32]. Wei’s study suggested that arsenic induced pancreatic ferroptosis through the activation of ferritinophage [31]. However, the mechanisms by which arsenic compounds induce ferroptosis in cancer cells remain to be fully elucidated.

The present study aimed to demonstrate that combination therapy has the potential to treat homologous recombination (HR)-proficient platinum-resistant OC. Thus, we tested the synergistic effect of combined PARPi with ATO in HR-proficient platinum-resistant OC in vitro and in vivo, and we further investigated the underlying mechanisms. Our findings provide novel mechanistic insights into the combined treatment of olaparib and ATO in platinum-resistant OC.

## Materials and methods

### Cell lines

Human cisplatin-resistant cell lines A2780-CIS and SKOV3-CIS were developed from A2780 and SKOV3 by chronic exposure to cisplatin. A2780-CIS and SKOV3-CIS cell lines were respectively cultured in RPMI 1640 and Dulbecco’s Modified Eagle’s Medium (DMEM) medium (BasalMedia, China) with 10% fetal bovine serum (FBS) (CellMax, SA211) and 1% penicillin/streptomycin. All cells were mycoplasma negative and incubated at 37 °C in 5% CO_2_.

### Primary cell culture

Tissue samples were collected from three patients diagnosed with high-grade serous OC at Women’s Hospital, Zhejiang University School of Medicine, China. The study was approved by the Hospital Ethical Committee (approval no. IRB-20220229-R). The samples were surgically removed and immediately processed as the following steps. Briefly, tissue samples were cut into several small pieces on ice and then digested using a mix of collagenase I, IV and DNase I (Sigma). Next, digested samples were filtered with a 40-μM cell strainer and then harvest the cells by centrifuging. After lysing the red blood cells (RBC) by RBC lysis buffer (Solarbio, R1010), the cells were harvested finally and resuspended in Advanced DMEM/F12 medium (Gibco, 12634028) containing 10% FBS.

### Reagents

Olaparib (HY-10162) was purchased from MedChemExpress (MCE) and prepared in dimethyl sulfoxide (DMSO) (Sigma, D2660) at 100 mmol/L and stored in aliquots in −80 °C. Carboplatin (HY-17393) was obtained from MCE and prepared in ultrapure water at 10 mmol/L and stored in aliquots in −80 °C. Ai-Ling#1 (ATO) solution (1 mg/ml) was obtained from Harbin Yida Pharmaceutical Co. Ferroptosis inhibitors Ferrostatin-1 (Fer-1, HY-100579), Liproxstatin-1 (Lip-1, HY-12726), Deferoxamine mesylate (DFO, HY-B0988), apoptosis inhibitor Z-VAD-FMK (HY-16658B), and oleic acid (OA, HY-N1446) were purchased from MCE. Compound C (CC, S7840) was purchased from Selleck Chemicals.

### Cytotoxicity, cell viability assay, and drug combination analysis

Cells were plated in 96-well plates at a density of 2000 cells per well for 24 h and treated with indicated agents. Then the cell viability and half-maximal inhibitory concentrations (IC_50_) were assessed using Cell Counting Kit-8 (CCK-8) assay (DOJINDO, CK04). The combination effect between olaparib and ATO was determined by CompuSyn software using Chou-Talalay method. Combination indexes (CI) <1 indicates synergistic effects.

### Colony formation assay

In all, 600 per well cells (SKOV3-CIS) or 1000 per well cells (A2780-CIS) were plated in 12-well plates for 24 h and then treated continuously with DMSO, olaparib, ATO, or olaparib in combination with ATO at indicated concentrations for 10 days. Cells were stained in the methanol containing 0.1% crystal violet, and then colonies were imaged were by a camera.

### Cell apoptosis measurement

Annexin V-fluorescein isothiocyanate (FITC)/propidium iodide (PI) apoptosis kit (Multi Sciences, AP101) was used to detect the percentage of cell apoptosis. Cells with appropriate density per well were incubated in 6-well plates for 24 h, followed by treatments with DMSO, olaparib, ATO or olaparib in combination with ATO for 48 h. All cells were resuspended in pre-chilled 1 × binding buffer, stained with FITC and PI for 5 min at room temperature (RT) in the dark and analyzed by flow cytometer (BD Biosciences, FACS Verse).

### Immunofluorescence

Cells were plated onto the autoclaved coverslips at an appropriate density per well 24 h before drug treatment. After fixation, permeabilization, and blocking, cells were probed with phosphor-histone H2AX (γ-H2AX) (Abcam, ab26350, 1:5000 dilution) at 4 °C overnight, washed with PBS and then stained with fluorescently conjugated secondary antibody (Invitrogen, A32723, 1:1000 dilution) for 1 h at RT in the dark. Finally, cells were counterstained with DAPI (Abcam, ab104139) and imaged under a confocal laser-scanning microscope (Olympus, FLUOVIEW FV1200). The γ-H2AX foci in each cell were captured and counted.

### SCD1 overexpression

The expression plasmid of SCD1 was constructed by GenePharma Biotech (Shanghai, China). Transfection of the expression plasmid was performed using X-tremeGENE™ HP DNA Transfection Reagent (Roche, 06366546001) according to the manufacturer’s instructions.

### RNA isolation and quantitative real-time PCR

Total RNA was extracted using TRIzol reagent (Invirogen, 15596018). mRNA was reverse transcribed into cDNA with HiScript III RT SuperMix for qPCR (+gDNA wiper) (Nanjing Vazyme Biotech, R323-01). ChamQ Universal SYBR qPCR Master Mix (Nanjing Vazyme Biotech, Q711-02) and the biosystems 7900HT Fast Real-Time PCR System (Life Technologies, Carlsbad, CA, USA) were used to perform the quantitative real-time PCR. The primers were presented as follows: human SCD1: 5′-TCTAGCTCCTATACCACCACCA-3′ (forward), 5′-TCGTCTCCAACTTATCTCCTCC-3′ (reverse), human β-actin: 5′-TGGTATCGTGGAAGGACTC-3′ (forward), 5′-AGTAGAGGCAGGGATGATG-3′ (reverse).

### Western blot analysis

After lysed with RIPA buffer (Solarbio, R0010), cell lysates were separated on SDS-PAGE gels and blotted onto membranes, then blocked with 5% milk for 1 h at RT and immunoblotted with primary antibodies at 4 °C overnight: γ-H2AX (Abcam, ab26350, 1:1000 dilution), caspase3 (Cell Signaling Technology, 9662, 1:1000 dilution), cleaved caspase3 (Cell Signaling Technology, 9664, 1:1000 dilution), β-actin (Fude Bio, FD0060, 1:5000 dilution), SLC7A11/xCT (Cell Signaling Technology, 126915, 1:1000 dilution), GPX4 (Abcam, ab125066, 1:1000 dilution), SCD1 (Abcam, ab236868, 1:1000 dilution), ACSL3 (Santa Cruz, sc-166374, 1:200 dilution), and Phospho-AMPKα (Thr172) (Cell Signaling Technology, 2535, 1:1000 dilution). Finally, the membranes were incubated with secondary antibodies conjugated to HRP (Fude Bio, 1:5000 dilution) and visualized by imaging systems.

### Single cell gel electrophoresis assay

Evaluation of cellular DNA damage was detected by a Comet assay kit (Abcam, ab238544). Briefly, treated cells were collected by gentle scraping and resuspended at 1 × 10^5^ per ml in PBS. After creating the comet base layer, mix cell suspension with comet agarose and immediately transfer the mixture onto the base layer. The cells were lysed for 1 h at 4 °C in the dark and then electrophoresis was performed for 30 mins at 16 volts, 300 mA. Then, the slides were washed with pre-chilled DI H_2_O and immersed in 70% pre-chilled ethanol. Finally, the air-dried slides were stained by Vista Green DNA Dye at RT for 15 mins and images were obtained by epifluorescence microscopy using a FITC filter (Leica CTR6500). DNA damage was expressed as “DNA in tails (%)” and the average percentage of DNA in tails was measured for 50 cells in each group via CometScore 2.1 Software (Tritek).

### Measurement of lipid peroxidation

Lipid peroxidation was analyzed by flow cytometry and confocal laser-scanning microscope. For flow cytometry analysis, cells were plated in six-well culture plate overnight and then treated with indicated agents. Next, cells were incubated with 10 μM BODIPY 581/591 C11 (Thermo Fisher, D3861) for 30 min, washed with PBS, trypsinized and resuspended in PBS, and then subjected to flow cytometry analysis. For confocal imaging, cells were seeded in a 4-chamber glass bottom dish (Cellvis, D35C4-20-1.5-N) and treated as previously indicated. After incubated with 5 μM BODIPY 581/591 C11 for 30 min and washed with PBS, images were acquired using the confocal laser-scanning microscope.

### Transmission electron microscopy

To visualize the cellular change of ultrastructural morphology, treated cells were firstly fixed with 2.5% glutaraldehyde for 1 day, then postfixed with 1% OsO4 for 1 h. Followed by the dehydration with 30%, 50%, 70 and 80% ethanol 90%, 95% acetone and absolute acetone in sequence, cells were embedded at RT for 4 h. Finally, the cells were embedded in Eppendorf contained Spurr resin, sectioned in LEICA EM UC7 ultratome and stained by uranyl acetate and alkaline lead citrate for 10 min respectively. Images were acquired under Hitachi Model H-7650 TEM.

### Malondialdehyde assay

After indicated treatments, cells were homogenized and quantified with the BCA protein assay kit. Cellular MDA content was determined using a Cell MDA assay kit (Nanjing Jiancheng Bioengineering Institute, A003-4-1).

### In vivo Xenograft mouse model

Xenograft mouse model experiments were conducted in accordance with a protocol reviewed and approved by the Animal Care Committee of Zhejiang Chinese Medical University (approval no. IACUC-20210823-10). Twenty-four female BALB/c nude mice (Shanghai Slack Laboratory Animal Co. Ltd, China) aged 5-week-old were housed at a specific pathogen-free facility in the animal research center of Zhejiang Chinese Medical University. The subcutaneous mouse model of platinum-resistant OC was established by injecting SKOV3-CIS cells (1 × 10^7^ cells in 0.1 ml PBS) into the left flank of each mouse. When the tumor size reached ~100 mm^3^, mice were randomly assigned to 4 groups: (1) control (200 μl of solvent intraperitoneally); (2) olaparib (50 mg/kg/day intraperitoneally); (3) ATO (2.5 mg/kg/day intraperitoneally); and (4) the combination group (olaparib 50 mg/kg/day plus ATO 2.5 mg/kg/day intraperitoneally). The agents were administrated for 20 days. The formula, volume = 1/2 (length × width^2^), was used to calculate the tumor volumes every 4 days. After 20-day treatment, all the mice were killed, the tumors and organs (heart, liver, spleen, lung, and kidneys) were isolated and stored in 4% paraformaldehyde, blood sample from each mouse was measured the biochemical and routine blood testing indexes to test the biosafety of the combination therapy.

### Immunohistochemistry

The dissected tumors and organs were paraffin embedded and cut into sections for HE staining. For immunohistochemistry (IHC) staining, the tumor sections were deparaffinized, hydrated, antigen retrieved, and then incubated with the primary antibody against Ki-67 (Abcam, ab15580, 1:100 dilution), cleaved caspase3 (Cell Signaling Technology, 9661, 1:400 dilution), SCD1 (Abcam, ab236868, 1:1000 dilution), and 4-HNE (Abcam, ab46545, 1:100 dilution) followed by the incubation with the secondary antibodies for 1 h in the next day.

### Statistical analysis

All experiments were routinely performed using at least three biological replicates and independently repeated for at least three times. The combination effect was determined by CompuSyn software using Chou-Talalay method and represented by heatmap using the pheatmap package of R Studio (Version 4.1.0). The data were represented as mean ± standard deviation (SD) and analyzed with Graphpad Prism 9.0 and SPSS 26.0 Software. Differences were considered to be statistically significant at *p < 0.05, **p < 0.01 and ***p < 0.001.

## Results

### ATO plus olaparib exerts synergistic cytotoxicity in platinum-resistant OC cells

**Fig. 1:**
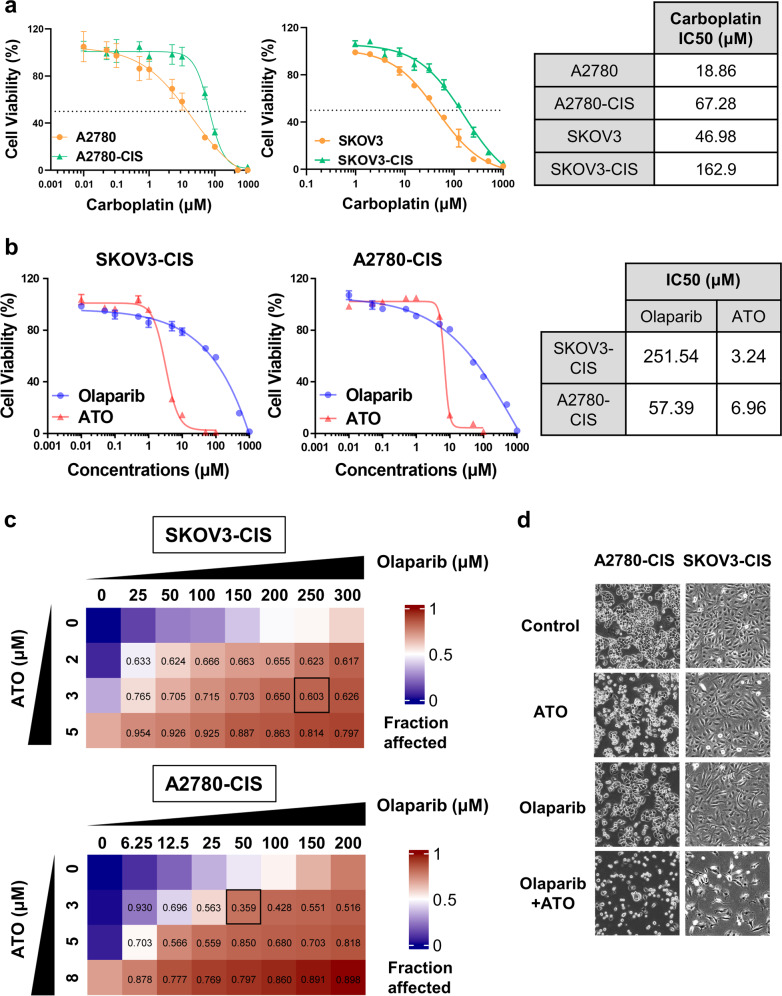
ATO plus olaparib exerts synergistic cytotoxicity in platinum-resistant OC cells. **a**, **b** Drug-response curves of survival and IC_50_ values for cell viability after a series of concentrations treatment of carboplatin (**a**), olaparib or ATO (**b**) in the ovarian cancer cell lines measured by CCK-8 assay at 72 h. **c** Representative synergy matrix heatmaps for olaparib and ATO treatment in SKOV3-CIS and A2780-CIS cells. Cells were exposed to the indicated concentrations of olaparib and ATO for 72 h and cell viabilities were assessed by a CCK-8 assay. The combination index (CI) values were determined by CompuSyn software and shown numerically inside the boxes of the heatmaps. CI values < 1 indicate synergistic effects. **d** After treatment with 250 μM olaparib and 3 μM ATO in SKOV3-CIS cells, 50 μM olaparib and 3 μM ATO in A2780-CIS cells for 48 h, the morphology alteration was observed using an inverted phase contrast microscope (Leica CTR6500). Error bars are represented as mean ± SD from three independent repeats.

At present, carboplatin is used more often than cisplatin as the first-line therapeutic agent in the treatment of OC, due to its low toxicity profile compared with cisplatin [35–37]. Therefore, carboplatin resistance was investigated in the following two paired cell lines: A2780, A2780-CIS and SKOV3, SKOV3-CIS. The IC_50_ was 3.56-fold higher in A2780-CIS than that in A2780 cells (67.28 μM vs. 18.86 μM), and 3.47-fold higher in SKOV3-CIS than that in SKOV3 cells (162.9 μM vs. 46.98 μM; Fig. 1a). To evaluate the combined effect of olaparib and ATO in platinum-resistant OC, the two platinum-resistant cells were subjected to subsequent studies.

To explore olaparib and ATO-induced cytotoxicity in platinum-resistant OC cells, the loss of cell viability was assessed using CCK-8 assays in SKOV3-CIS and A2780-CIS cells. Results of the CCK-8 assay indicated that either olaparib or ATO inhibited the viability of both cell lines in a dose-dependent manner. The IC_50_ of olaparib and ATO in SKOV3-CIS and A2780-CIS cells were presented in Fig. 1b. To investigate the synergistic effects of olaparib combined with ATO, the cell viability of A2780-CIS and SKOV3-CIS cells were determined, following treatment with various concentrations of olaparib in combination with ATO. The combination index (CI) values were determined using CCK-8 assays and Compusyn software (Fig. 1c). The synergetic effects (CI < 1) occurred in all indicated drug combinations. For A2780-CIS cells, the combination of 50 μM olaparib with 3 μM ATO demonstrated a notable effect on cell viability, with a CI value of 0.359. For SKOV3-CIS cells, the optimal synergetic effect was obtained when 250 μM olaparib was combined with 3 μM ATO, with a CI value of 0.603. Therefore, the indicated concentrations of each drug were used in subsequent experiments. The cell morphological changes were confirmed using light microscopy (Fig. 1d).

### ATO synergistically enhances olaparib-mediated cytotoxicity in platinum-resistant OC cells by inhibiting cell proliferation and promoting cell apoptosis

**Fig. 2:**
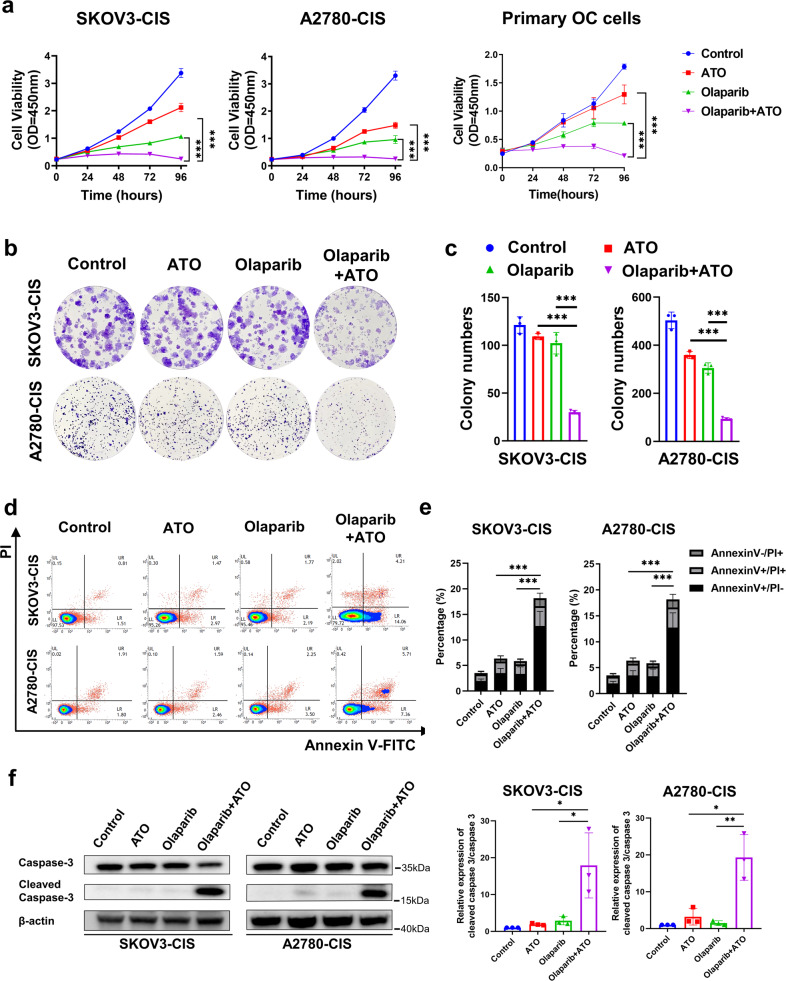
ATO synergistically enhances olaparib-mediated cytotoxicity in platinum-resistant OC cells by inhibiting cell proliferation and promoting cell apoptosis. **a** The 96 h cellular proliferation curves of SKOV3-CIS (left), A2780-CIS (middle), and primary OC cells (right) treated with olaparib (250 μM for SKOV3-CIS, 50 μM for A2780-CIS), ATO (3 μM) monotherapy or their combination, detected by CCK-8 assays. Error bars of the cell lines are represented as mean ± SD from three independent repeats. Error bars of the collated data from the three primary OC cells are presented as mean ± SD. **b** Representative images of the colony formation after treatment with DMSO, olaparib (2.5 μM for SKOV3-CIS, 1 μM for A2780-CIS), ATO (0.2 μM for SKOV3-CIS, 1 μM for A2780-CIS), or olaparib in combination with ATO. **c** Quantification of colony numbers of SKOV3-CIS and A2780-CIS cells subjected to the indicated treatments. **d**, **e** SKOV3-CIS and A2780-CIS cells were treated with DMSO, olaparib (250 μM for SKOV3-CIS, 50 μM for A2780-CIS), ATO (3 μM) or their combination for 48 h. The representative results (**d**) and histogram of apoptotic rates (**e**) were shown after Annexin V-FITC/PI staining. **f** The expression of cleaved caspase3 and caspase3 after indicated treatments monitored by western blot, The protein expression levels were normalized with β-actin. The normalized value of the control group was set to 1. Error bars are shown as mean ± SD from three independent repeats.

To determine the inhibitory effects of olaparib, ATO or the combined treatment of both, cells were treated with the indicated concentrations continuously for 96 h, and CCK-8 assays were performed. As demonstrated in Fig. 2a, combined treatment using olaparib with ATO abrogated the growth of SKOV3-CIS and A2780-CIS cells, compared with the single-agent treatment. We also investigated the inhibitory effects of the combined treatment in primary OC cells. Both olaparib and ATO slightly inhibited the growth of the primary OC cells while the combination of olaparib and ATO significantly suppressed the growth of the primary OC cells, compared to the single-agent treatment (Fig. 2a). Additionally, the colony formation assays were carried out to assess the anti-proliferation effects of olaparib in combination with ATO. Due to long-term drug exposure during colony formation assays, lower concentrations of olaparib and ATO were used as indicated in Fig. 2b, c, and the results demonstrated a significant reduction of formed colonies in the combination groups, compared with groups treated with each drug alone.

Annexin V-FITC/PI dual staining assays were performed to quantify the effects of each drug on cell apoptosis. The number of apoptotic A2780-CIS and SKOV3-CIS cells was increased following treatment with the combination of olaparib and ATO, compared with single drug treatment in each cell line (Fig. 2d, e). The ratio of cleaved caspase3 and caspase3, the apoptosis marker, was also detected using western blot analysis. The results demonstrated that single-agent slightly increased the ratio of cleaved caspase3 and caspase3, but the treatment of combined olaparib with ATO significantly increased the ratio of cleaved caspase3 and caspase3 in both SKOV3-CIS and A2780-CIS cells (Fig. 2f).

In summary, a combination of olaparib and ATO was synergistic in decreasing cell survival and colony formation, and promoting cell apoptosis in platinum-resistant OC cells.

### Olaparib and ATO synergistically promote cell apoptosis by increasing DNA damage in platinum-resistant OC cells

As olaparib and ATO cause DNA damage, we hypothesized that the combined treatment would further enhance the effects of DNA damage to promote cell apoptosis. Previous studies indicated that γ-H2AX was a marker of early DNA DSBs [37]. Thus, the levels of DNA damage were determined by the number of γ-H2AX-positive foci in each cell, γ-H2AX protein expression levels and a comet assay after indicated treatment.

**Fig. 3:**
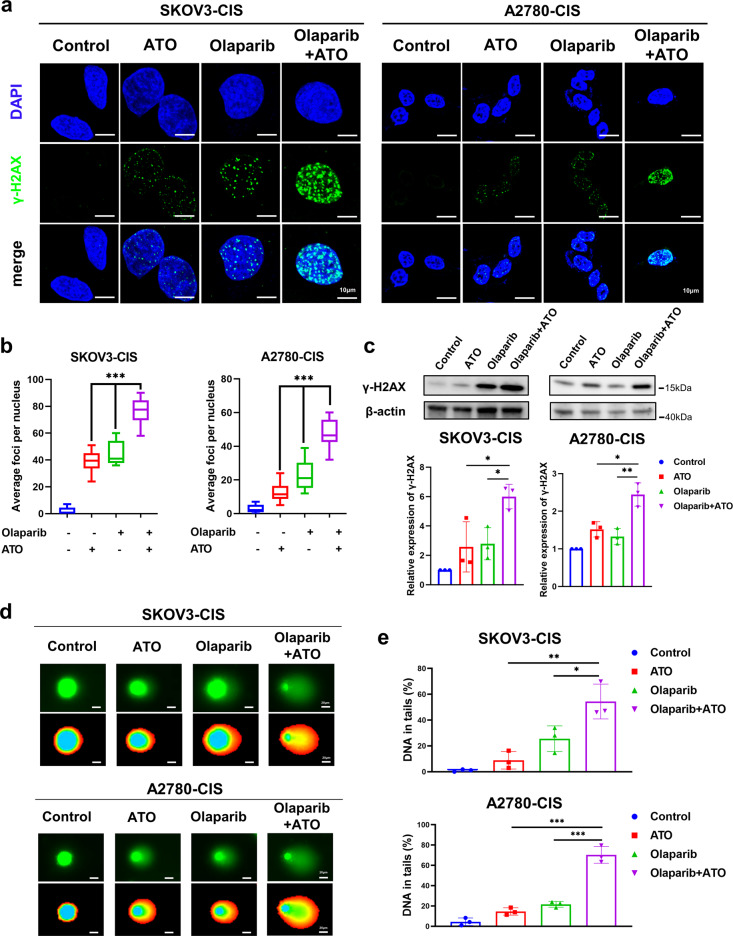
Olaparib and ATO synergistically promote cell apoptosis by increasing DNA damage in platinum-resistant OC cells. SKOV3-CIS and A2780-CIS cells were treated with DMSO, olaparib (250 μM for SKOV3-CIS, 50 μM for A2780-CIS), ATO (3 μM) or their combination for 48 h. **a** Representative immunofluorescence images of γ-H2AX foci in cells after indicated treatment. Magnification is ×100, scale bar = 10 μm. **b** Quantification of the number of γ-H2AX-positive foci in each nucleus based on immunofluorescence in SKOV3-CIS and A2780-CIS cells subjected to indicated treatments. Data was presented as mean ± SD from 50 cells. **c** Protein expression of γ-H2AX in cells after indicated treatment, detected using western blot. The protein expression levels were normalized with β-actin. The normalized value of the control group was set to 1. **d** Representative images DNA damage in SKOV3-CIS and A2780-CIS cells after different treatment, measured by comet assay, scale bar = 20 μm. **e** Quantified results by the tail moment in the comet assay obtained from 50 cells in each group. Error bars are shown as mean ± SD from three independent repeats.

As shown in Fig. 3a, b, immunofluorescence analysis demonstrated that the number of γ-H2AX-positive foci in each cell was increased following single drug treatment of olaparib or ATO, but were markedly accumulated when cells were subjected to the combined treatment of olaparib and ATO. Moreover, the results of Western blot assays suggested that the combination of both drugs significantly enhanced the protein expression levels of γ-H2AX, compared with the single-agent treatment (Fig. 3c). Results of the comet assay further demonstrated that olaparib induced more DNA in the tails when combined with ATO (Fig. 3d, e).

### Combined treatment of olaparib and ATO induces ferroptosis in platinum-resistant OC cells

**Fig. 4:**
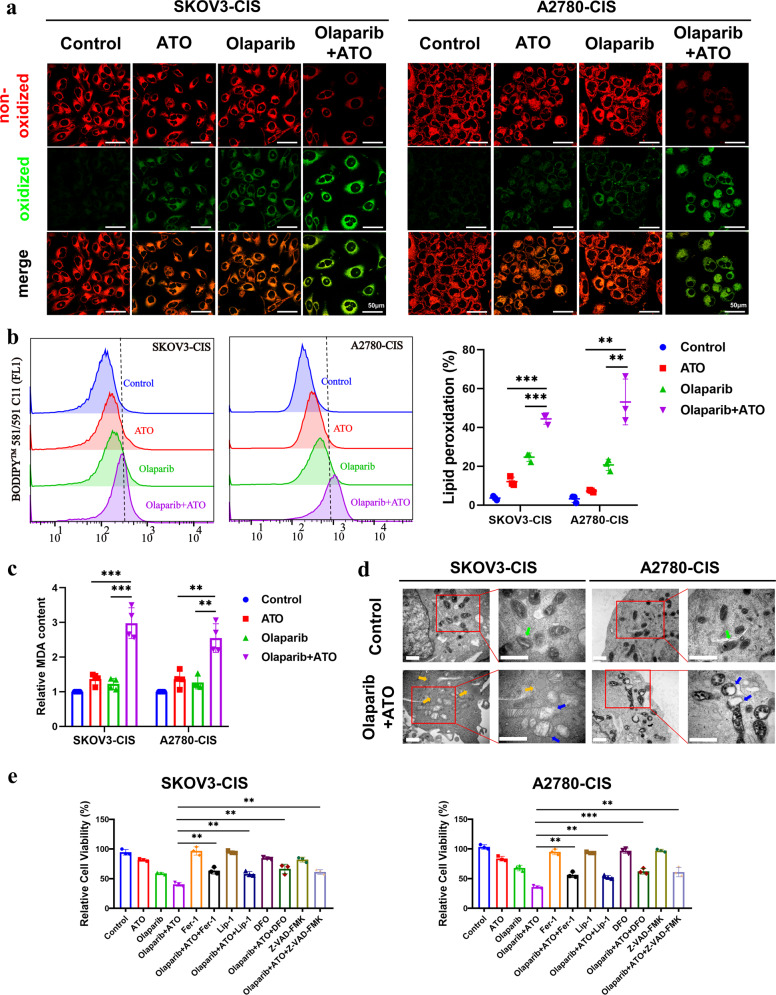
Combined treatment of olaparib and ATO induces ferroptosis in platinum-resistant OC cells. SKOV3-CIS and A2780-CIS cells were expose to DMSO, olaparib (250 μM for SKOV3-CIS, 50 μM for A2780-CIS), ATO (3 μM) or their combination for 48 h. **a** Representative confocal images of oxidized lipid reactive oxygen species (ROS) (green color) and reduced lipid peroxidation (red color) formation in SKOV3-CIS and A2780-CIS cells after different treatment. Magnification is ×60, scale bar = 50 μm. **b** Detection of the cellular lipid peroxidation level with BODIPY^TM^ 581/591 C11 determined by the flow cytometer. **c** Changes in intracellular MDA levels after indicated treatments, measured by an MDA Assay Kit. **d** Ultrastructural features of combinative treatment of olaparib and ATO treated SKOV3-CIS and A2780-CIS cells by TEM. The green arrows manifested the normal mitochondria and blue arrows showed the abnormal mitochondria with decreased mitochondrial cristae, orange arrows indicated the lipid formation. Scale bar = 1 μm. **e** Cell viability of SKOV3-CIS (left) and A2780-CIS (right) cells following olaparib and/ or ATO treatment in the presence or absence of Fer-1 (2 μM), Lip-1 (200 nM), DFO (10 μM), and Z-VAD-FMK (10 μM) for 48 h. Error bars are shown as mean ± SD from three independent repeats.

Ferroptosis has been demonstrated to contribute to olaparib-induced cytotoxicity in platinum-sensitive OC cells, and participate in pancreatic dysfunction induced by ATO [25, 31]. Thus, the role of ferroptosis in these synergistic effects was investigated in the present study. As ferroptosis is driven by the accumulation of lipid peroxidation products, lipid peroxidation was initially detected using confocal imaging, and the level of lipid peroxidation was quantified using flow cytometry. As demonstrated in Figs. 4a, b and S1, following the combined treatment of olaparib and ATO, lipid peroxidation was markedly increased in both the cell lines and the primary OC cells, compared with that of cells following single drug treatment. Consistently, malondialdehyde (MDA), the final product of lipid peroxidation, was also markedly increased in A2780-CIS and SKOV3-CIS cells following combined treatment (Fig. 4c). Meanwhile, an increased density of mitochondrial membrane, a decreased volume of cristae and several lipid droplets were observed in the cells treated with combined olaparib and ATO (Fig. 4d). Furthermore, it was observed that both the ferroptosis inhibitors (Fer-1, Lip-1, and DFO) and apoptosis inhibitor (Z-VAD-FMK) partly rescued the suppressed cell viability by combination of olaparib and ATO (Fig. 4e), which further confirmed that the combination treatment of olaparib and ATO triggered ferroptosis and promoted apoptosis in platinum-resistant OC cells.

**Fig. 5:**
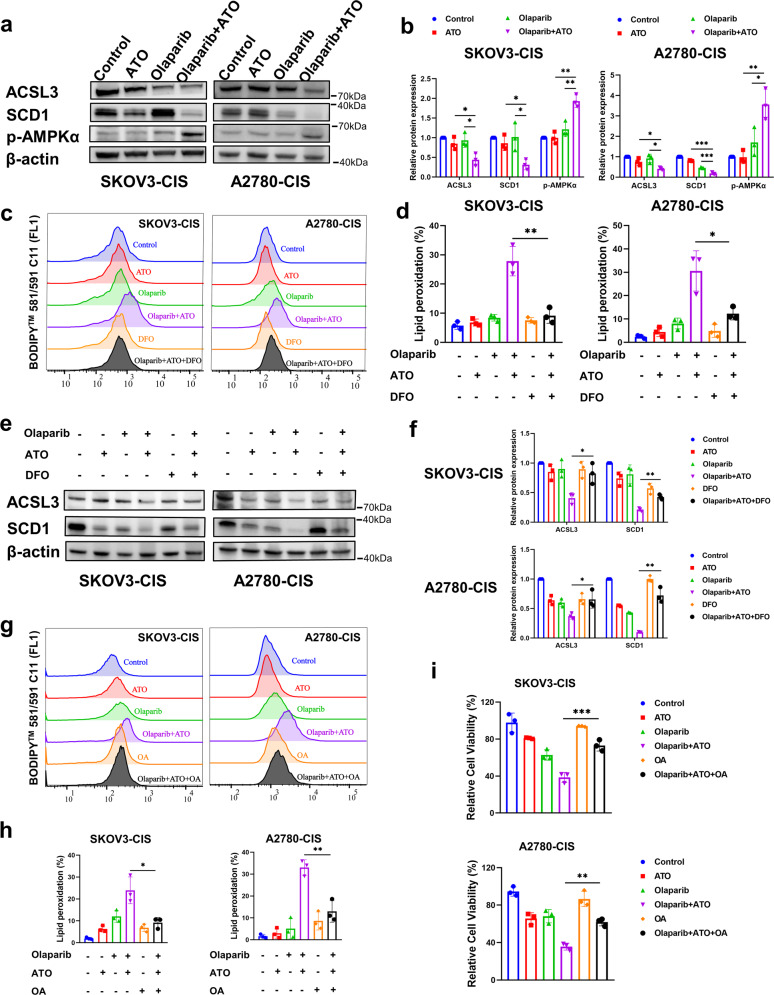
Combined treatment of olaparib and ATO triggered ferroptosis by activating AMPKα-SCD1 signaling. **a**, **b** Western blot to analyze the protein changes of ACSL3, SCD1, and p-AMPKα caused by the combined treatment. The protein expression levels were normalized with β-actin. The normalized value of the control group was set to 1. **c**–**f** Relative lipid peroxidation levels (**c**, **d**) and protein expression levels of ACSL3, SCD1 (**e**, **f**) in SKOV3-CIS and A2780-CIS cells following treatment of olaparib and/ or ATO in the presence or absence of 10 μM DFO. **g**–**i** Relative lipid peroxidation levels (**g**, **h**) and cell viability (**i**) in SKOV3-CIS and A2780-CIS cells following treatment of olaparib and/or ATO in the presence or absence of 50 μM OA. Error bars are shown as mean ± SD from three independent repeats.

To further investigate how the drug combination led to ferroptosis, several ferroptosis-related proteins (SLC7A11, GPX4, ACSL3, SCD1) were detected using western blot. Results of the present study demonstrated that ATO decreased the expression levels of SLC7A11 and phospholipid hydroperoxide glutathione peroxidase (GPX4), but no further reduction was found following the combined therapy (Fig. S2). Based on the theory that ferroptosis requires phospholipid peroxidation [38], and following the observed lipid droplets in present study, ferroptosis as a result of modulating cellular lipid metabolism was investigated. Moreover, the phosphorylation of AMPK *α* (p-AMPK *α*) was determined using western blot. Notably, in A2780-CIS and SKOV3-CIS cells, single agent treatment of olaparib or ATO upregulated the expression levels of p-AMPK *α* at low levels, and the combined treatment markedly amplified this phenomenon (Fig. 5a). The peroxidation of PUFAs is an important step in promoting ferroptosis, and monounsaturated fatty acids (MUFAs) contribute to ferroptosis resistance by competitively affecting the activity of PUFAs, which relies on acyl-CoA synthetase long-chain family member 3 (ACSL3) or stearoyl-CoA desaturase 1 (SCD1). As shown in Fig. 5a, the protein expression levels of SCD1 were slightly changed following single-agent treatment with ATO or olaparib in SKOV3-CIS and A2780-CIS cells. However, the combined treatment of olaparib and ATO greatly reduced the expression levels of SCD1 and ACSL3. In addition, as demonstrated in Fig. 5c, d, ferroptosis inhibitor DFO reversed lipid peroxidation caused by the combined treatment. Consistently, western blot also showed elevated expression of SCD1 and ACSL3 following the addition of DFO (Fig. 5e, f). These results suggested that combined olaparib and ATO triggered ferroptosis, and the underlying mechanism may be attributed to the activation of AMPK *α*-SCD1 signaling.

In complement to the mechanistic characterizations mentioned above, we also investigated the role of AMPK *α* in the combination-triggered ferroptosis in A2780-CIS and SKOV3-CIS cells by using Compound C (CC), which is an AMPK pathway inhibitor. It was detected that the addition of CC significantly rescued platinum-resistant OC cells from the enhanced lipid peroxidation (Figs. 3b and S3a) as well as the cytotoxic effects (Fig. S3c) caused by the combination treatment of olaparib and ATO.

SCD1 is an enzyme that converts saturated fatty acid to MUFAs, which was reported as a downstream of AMPK pathway [39]. We constructed SCD1 plasmid to overexpress SCD1 and verified the significantly upregulated expression of SCD1 by conducting RT-qPCR and Western blot in both SKOV3-CIS and A2780-CIS cells (Fig. S3d, e). It was found that SCD1 overexpression partially rescued the suppressed cell viability induced by the combination of olaparib and ATO (Fig. S3f). Furthermore, supplementation of MUFA oleic acid (OA), the primary catalytic products of SCD1 partly rescued the lipid peroxidation (Fig. 5g, h) promoted by the combination of olaparib and ATO as well as the inhibition effects on cell viability (Fig. 5i).

### Olaparib and ATO synergistically inhibit the tumor growth of SKOV3-CIS-derived xenograft models

**Fig. 6:**
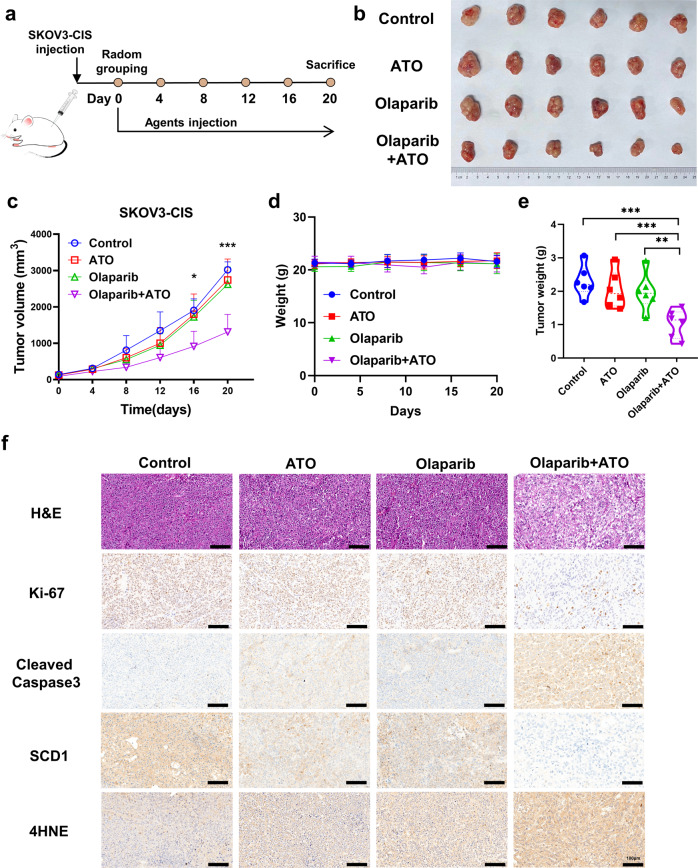
Olaparib and ATO synergistically inhibit the tumor growth of SKOV3-CIS-derived xenograft models. **a** Schematic illustrating the SKOV3-CIS mouse xenograft experimental design. SKOV3-CIS cells were implanted subcutaneously and grown until tumors reached ~100 mm^3^. Xenografted mice were randomized and then treated intraperitoneally with vehicle, olaparib (50 mg/kg), ATO (2.5 mg/kg), or the combination of both agents as indicated for continuously 20 days (*n* = 6 mice per group). **b** Tumor images collected from animals treated with vehicle, olaparib, ATO, or the combined agents at experimental endpoint. **c**, **d** Tumor volume and mice weight growth curves after indicated treatments at different time points. **e** Quantification of tumor weight at the experimental endpoint in each group. **f** Representative images of HE staining and IHC staining of Ki-67, cleaved caspase3, SCD1 and 4HNE for xenograft tumor sections. Error bars are represented as mean ± SD from six independent repeats. Scale bar, 100 μm.

To further explore the effects of olaparib in combination with ATO in platinum-resistant OC cells in vivo, SKOV3-CIS-derived subcutaneous xenograft tumors were established in nude mice. As shown in Fig. 6a, mice were randomly assigned to one of four groups to receive olaparib, ATO or olaparib combined with ATO, when the average volume of tumors reached ~100 mm^3^. Following the indicated treatment for 20 days, single agent treatment exhibited few effects on tumor growth; however, the combination of olaparib and ATO treatment caused markedly suppressed tumor growth (Fig. 6b, c, e). Moreover, IHC analysis demonstrated lower expression of Ki-67 (a marker of cell proliferation) and SCD1, higher expression of cleaved caspase3 and 4-HNE (the major lipid peroxidation product [40]) after the combined treatment of olaparib and ATO. These evidence indicated that the combined treatment of olaparib and ATO inhibited tumor proliferation, enhanced apoptosis and ferroptosis, leading to tumor suppression in vivo (Fig. 6f).

**Fig. 7:**
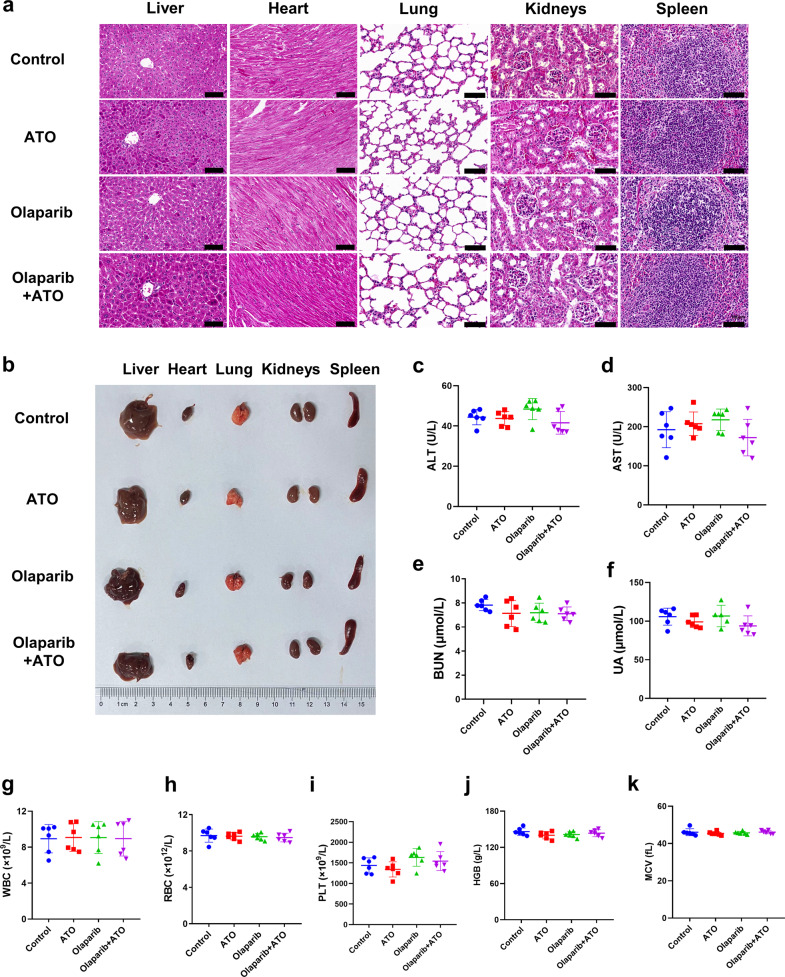
Preliminary biosafety evaluations of combination treatment of olaparib and ATO in mice model. **a**, **b** HE staining images and representative images of major organs (heart, liver, spleen, lung, and kidney) from mice after different treatments (Scale bar = 100 μm). **c**–**k** The blood biochemistry and hematology assays of mice treated with indicated agents at the endpoint. The testing parameters included examinations of alanine aminotransferase (ALT), aspartate aminotransferase (AST), blood urea nitrogen (BUN), uric acid (UA), red blood cell (RBC) counts, white blood cell (WBC) counts, platelets (PLT), hemoglobin (HGB), hematocrit (HCT), and mean corpuscular volume (MCV).

To examine the biosafety of the combination treatment of olaparib and ATO, changes in body weight, histological analysis, blood biochemistry, and hematologic biosafety studies were carried out in mice. Notably, no significant difference was found in the body weight of mice in the control or treatment groups (Fig. 6d). Additionally, the administration of olaparib in combination with ATO did not cause any morphological changes in major organs (Fig. 7a, b). Moreover, the blood biochemistry and hematologic examinations demonstrated the desirable biosafety of the combined treatment of olaparib and ATO (Fig. 7c–k).

## Discussion

Results of the present study highlighted that the PARPi, olaparib, in combination with ATO exerts synergistic cytotoxicity in platinum-resistant OC in vitro and in vivo. The in vitro studies confirmed that combined treatment of olaparib and ATO significantly suppressed cell proliferation, increased the single agent-induced DSBs and cell apoptosis, and triggered ferroptosis in platinum-resistant OC cells. The underlying mechanism is likely through AMPK*α*-SCD1 signaling, which plays an important role in cancer energy metabolism and ferroptosis [39, 41]. Consistently, the subsequent in vivo experiments demonstrated that this combination strategy exhibited synergistic suppression activity and satisfactory biosafety in mice.

The targeted therapies of PARPi have improved the current scope of OC treatment; however, PARPi monotherapy in patients with platinum-resistant OC exerted limited effects [18, 42, 43]. According to a phase II study, the objective response rate to PARPi were 64.9% and 23.4% in platinum-sensitive and platinum-resistant/refractory patients with a PFS of 9.4 and 7.2 months, respectively [43]. Thus, novel combination strategies are required to efficiently inhibit the growth and development of platinum-resistant OC cells. Recent studies have also demonstrated that the use of olaparib in combination with other agents exerted complementary mechanisms of cytotoxicity [44–46]. Our previous study highlighted the synergistic effects of PARPi and ATO in HR-proficient OC cells [47]. However, the effective combined treatment of olaparib in platinum-resistant OC is yet to be fully characterized. In the present study, the effectiveness of this drug combination in HR-proficient platinum-resistant OC cells was explored. Results of the present study demonstrated that treatment with olaparib and ATO was more effective in inhibiting the growth of platinum-resistant OC cells, and greatly increased the rate of cell apoptosis, compared with either drug treatment alone. The mechanism underlying olaparib-induced apoptosis includes the accumulation of SSBs and subsequent DSBs [13, 14]. Notably, ATO has been reported to inhibit the growth of leukemia and several solid tumors, such as osteosarcoma and lung carcinoma, by the accumulation of DNA damage [30, 48–50]. The present study determined the levels of DNA damage using a comet assay, γ-H2AX protein levels and the number of γ-H2AX-positive foci in each cell. Consistent with our previous data and the work of other research groups, the addition of ATO significantly increased olaparib-induced DSBs and promoted cell apoptosis [47].

As the results of a previous study which demonstrated that olaparib promoted OC cell death by enhancing ferroptosis in parallel to DNA damage-mediated cell apoptosis [25], the present study further investigated whether ferroptosis contributed to the combined effectiveness of olaparib and ATO. Results of the present study demonstrated a significant accumulation of lipid peroxidation and mitochondrial morphologic change following the combined treatment of olaparib and ATO in platinum-resistant OC cells. Results from a previous study indicated that SLC7A11 participated in the olaparib-induced ferroptosis in OC cells [25]. Additionally, ATO was reported to induce pancreatic dysfunction and ferroptosis via reducing the expression of GPX4 [31]. Therefore, the protein expression levels of SLC7A11 and GPX4 were initially explored in the present study, and the results revealed that ATO suppressed the expression levels of both SLC7A11 and GPX4, but the combined treatment exhibited no significant further effects. It is likely that multiple mechanisms contribute to ferroptosis, including iron accumulation, dysfunction of lipid metabolism, and the antioxidant system’s inactivation [38].

Furthermore, results of a previous study indicated that olaparib may increase AMPKα activity and the subsequent downstream transcription [51]. Based on previous reports and the lipid droplets observed in the transmission electron microscopy (TEM) assays in the present study, we hypothesized that the combined treatment-induced ferroptosis was regulated by lipid metabolism-associated signaling. The phosphorylation of the energy sensor, AMPKα, was significantly upregulated following the combined treatment in the present study, and the results were further confirmed with AMPK inhibitor CC while CC partly restored the increased lipid peroxidation and inhibited cell viability caused by the combined treatment. MUFAs inhibit ferroptosis, and depend on either ACSL3 or SCD1 [52]. Results of a previous study suggested that ACSL3-activated exogenous MUFAs can displace PUFAs located at the plasma membrane and make them resistant to ferroptosis [52]. SCD1 is a lipid modifying enzyme that catalyzes the desaturation of saturated fatty acids, and it is notably upregulated in numerous malignancies, such as bladder, breast, liver as well as ovarian cancer [53]. Thus, SCD1 may protect multiple cancer cells from ferroptosis, including OC cells [54–57]. Results of the present study highlighted a significant decrease of both ACSL3 and SCD1 following the combined treatment of olaparib and ATO. Moreover, ferroptosis inhibitor DFO partly rescued the reduced protein expression levels of ACSL3 and SCD1 as well as the suppressed accumulated lipid peroxidation. Furthermore, the addition of OA, the primary catalytic products of SCD1, also partly restored the inhibited cell viability and the elevated lipid peroxidation by the combined treatment. Collectively, these results confirmed that the combined treatment of olaparib and ATO triggered ferroptosis, and the results further demonstrated that this treatment is associated with lipid peroxidation and ferroptosis through an unrecognized mechanism, which is likely to involve the repression of SCD1.

The Annexin V-FITC/PI dual staining assay was widely used to determine the cell apoptosis fraction. The Annexin V + / PI– fraction represents apoptotic cells while Annexin V + / PI + may include both late apoptotic and necrotic cells. Our data, together with previous studies [39, 56, 58–61], demonstrated a subset of cells with Annexin V-/ PI + in ferroptosis, detected by Annexin V/PI assay, and our subsequent results further verified the co-treatment-induced ferroptosis. However, some studies indicated that cells with Annexin V + / PI + represented the initiation process of ferroptosis, which needs to perform time-course experiments to elucidate. The time of the co-treatment triggered ferroptosis will be explored in our future studies.

Since the high heterogeneity of OC, appropriate cell lines to study high-grade serous OC are challenging. Despite the extensive use of A2780 and SKOV3 cells in preclinical research, we must acknowledge that a limitation in the present study is that SKOV3-CIS and A2780-CIS cells were not the appropriate choice to study high-grade serous OC [62]. To complementally demonstrate the synergistic effects, we investigated the inhibition effects of the combination in primary OC cells using CCK-8 assay and detected the lipid peroxidation with BODIPY 581/591 C11 determined by the flow cytometer as well. The results further confirmed the synergistic effects and enhanced lipid peroxidation in OC cells following the combined treatment.

In conclusion, results of the present study revealed that olaparib, in combination with ATO, exerted synergistic cytotoxicity in platinum-resistant OC in vitro, and suppressed tumor growth at a higher level than either agent alone in vivo. Mechanistically, the combined treatment of olaparib and ATO promoted apoptosis by an accumulation of DNA damage and triggered ferroptosis, which may depend on the AMPK *α*-SCD1 signaling pathway.

## Supplementary information

Original Data File

Supplementary Materials

Reproducibility Checklist

## Data Availability

All data needed to evaluate the conclusions in the paper are present in the paper and/or the Supplementary Materials. Additional data are available from the corresponding authors on reasonable request.
