# Supplementary material for: Olaparib synergizes with arsenic trioxide by promoting apoptosis and ferroptosis in platinum-resistant ovarian cancer
Source: Cell Death Dis. 2022 Sep 27;13(9):826. doi: 10.1038/s41419-022-05257-y (PMC9513087; doi:10.1038/s41419-022-05257-y)
Supplement: Supplementary file 1 — Original Data File [file 41419_2022_5257_MOESM1_ESM.pptx]

## Slide 1
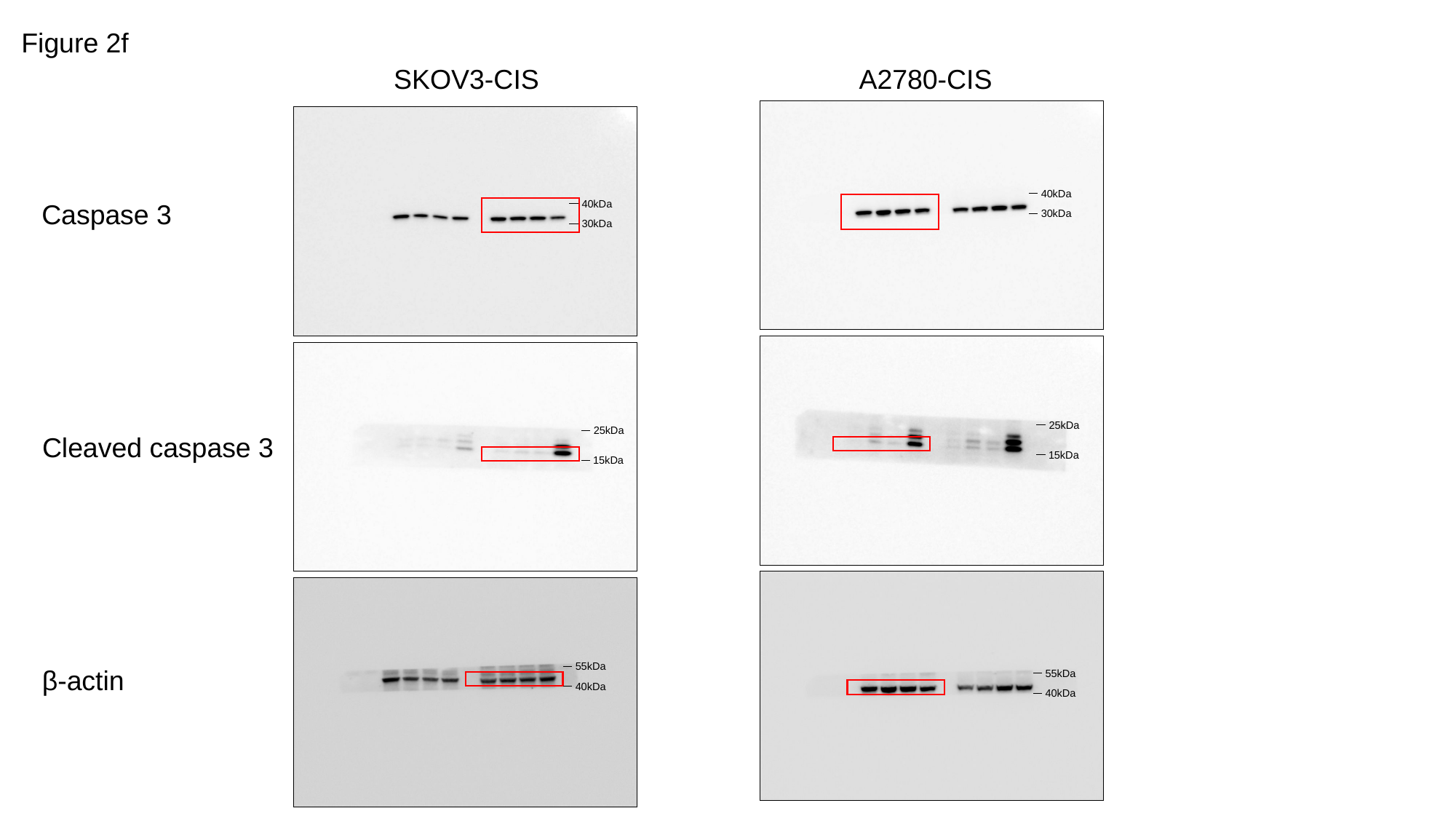

Figure 2f
SKOV3-CIS
A2780-CIS
40kDa
30kDa
40kDa
30kDa
Caspase 3
25kDa
15kDa
25kDa
15kDa
Cleaved caspase 3
55kDa
40kDa
β-actin
55kDa
40kDa

## Slide 2
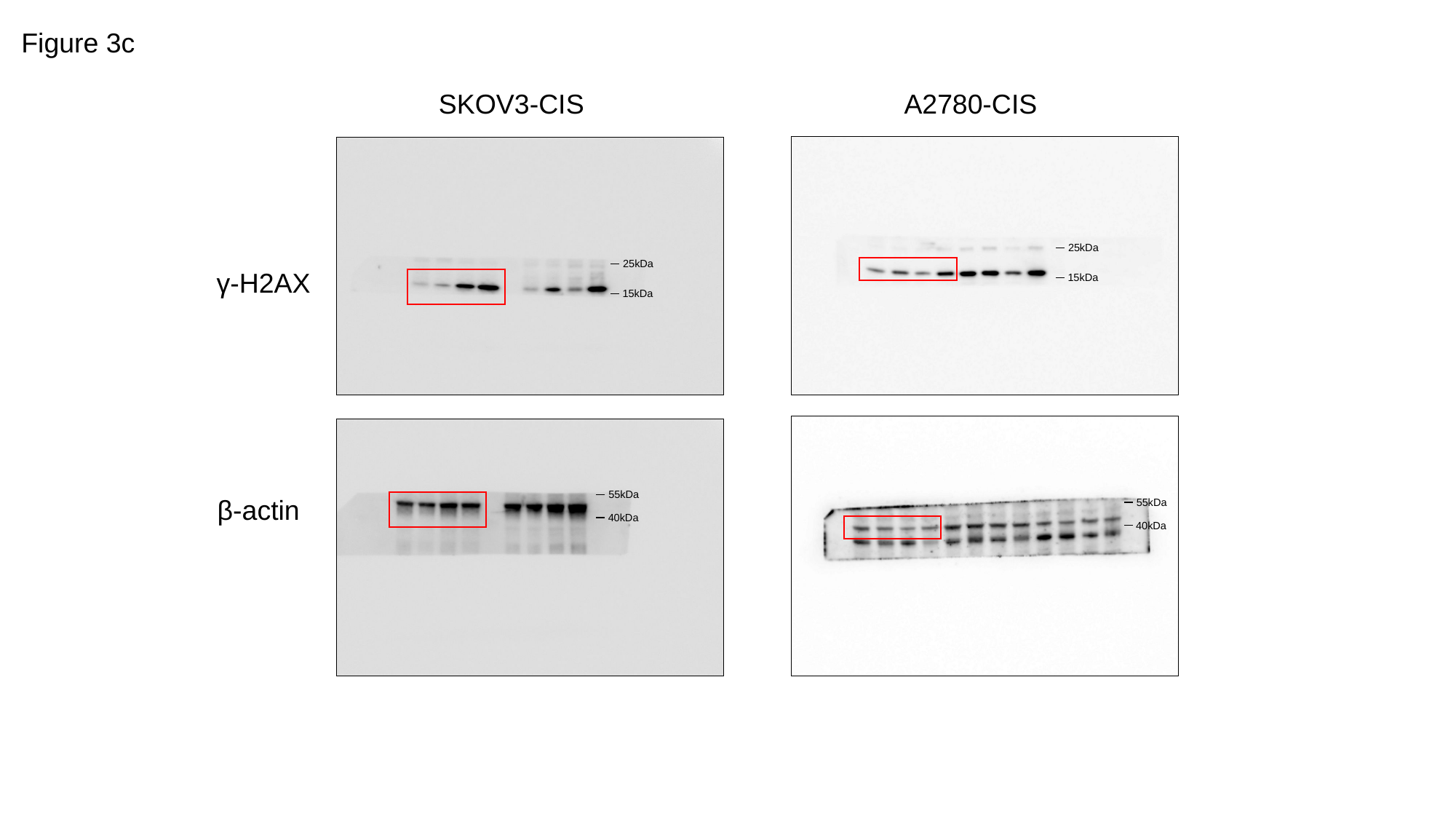

Figure 3c
SKOV3-CIS
A2780-CIS
25kDa
15kDa
25kDa
15kDa
γ-H2AX
55kDa
40kDa
β-actin
55kDa
40kDa

## Slide 3
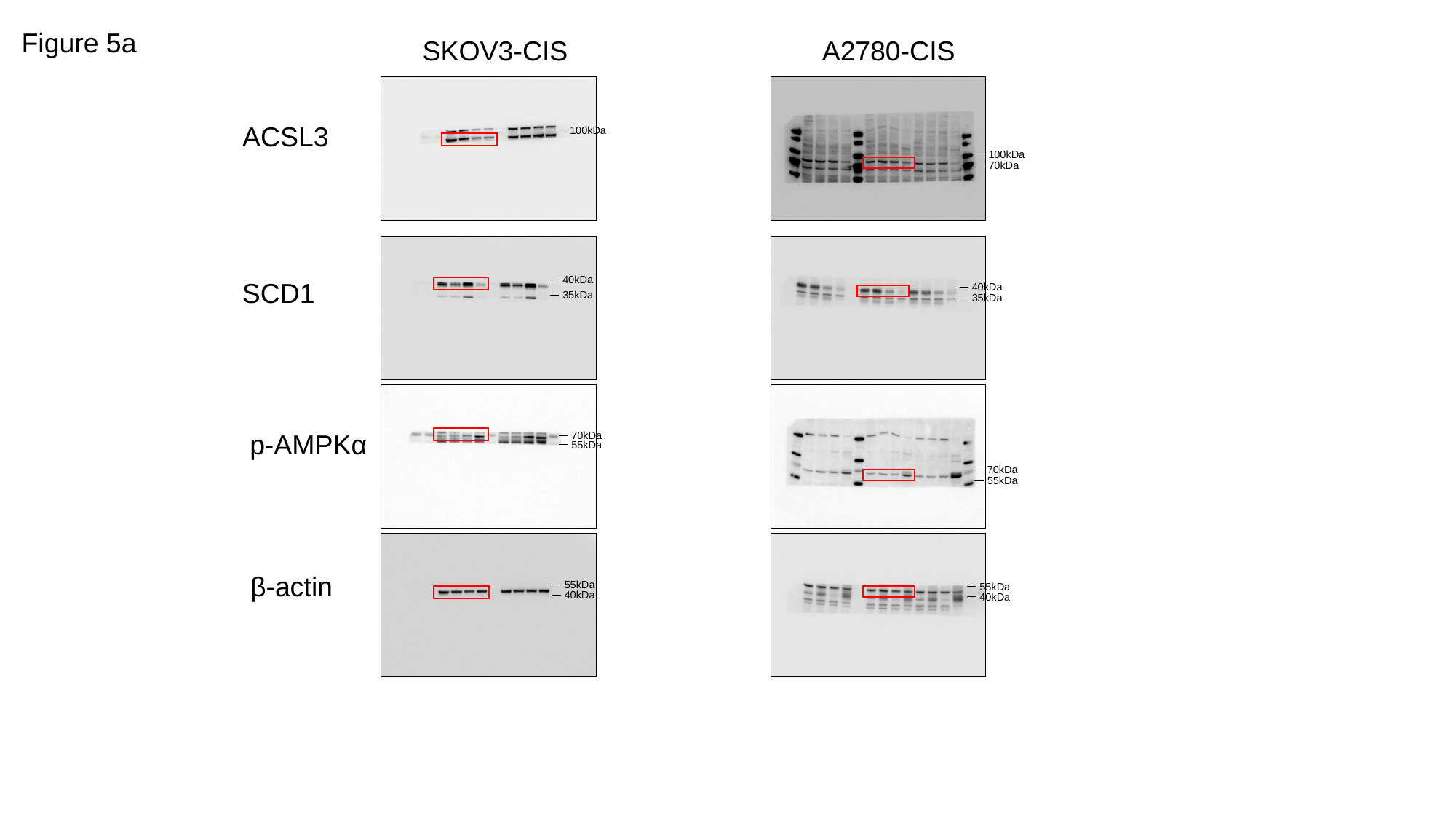

Figure 5a
SKOV3-CIS
A2780-CIS
ACSL3
100kDa
100kDa
70kDa
40kDa
35kDa
SCD1
40kDa
35kDa
p-AMPKα
70kDa
55kDa
70kDa
55kDa
β-actin
55kDa
40kDa
55kDa
40kDa

## Slide 4
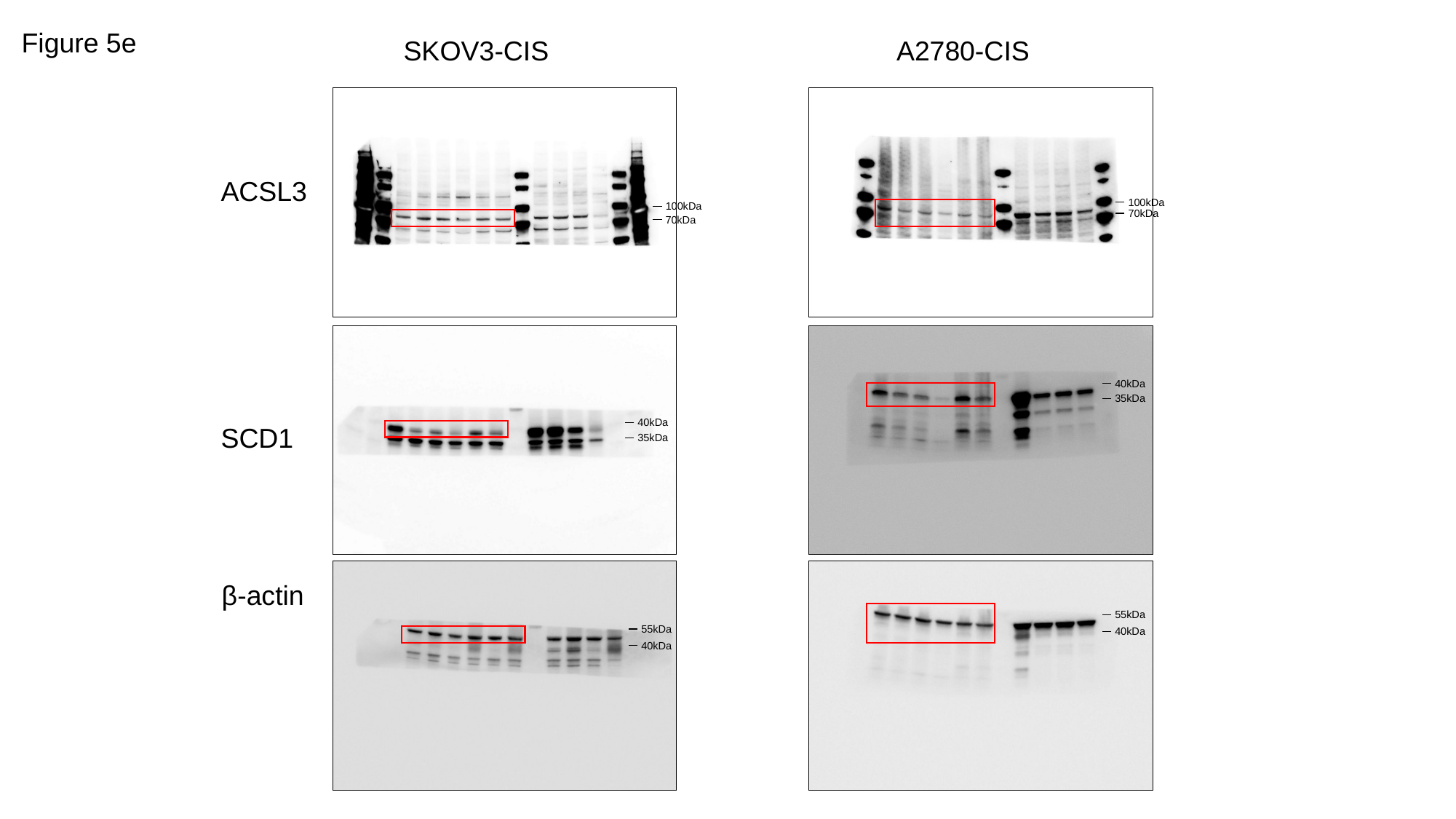

Figure 5e
SKOV3-CIS
A2780-CIS
ACSL3
100kDa
70kDa
100kDa
70kDa
40kDa
35kDa
40kDa
35kDa
SCD1
β-actin
55kDa
40kDa
55kDa
40kDa

## Slide 5
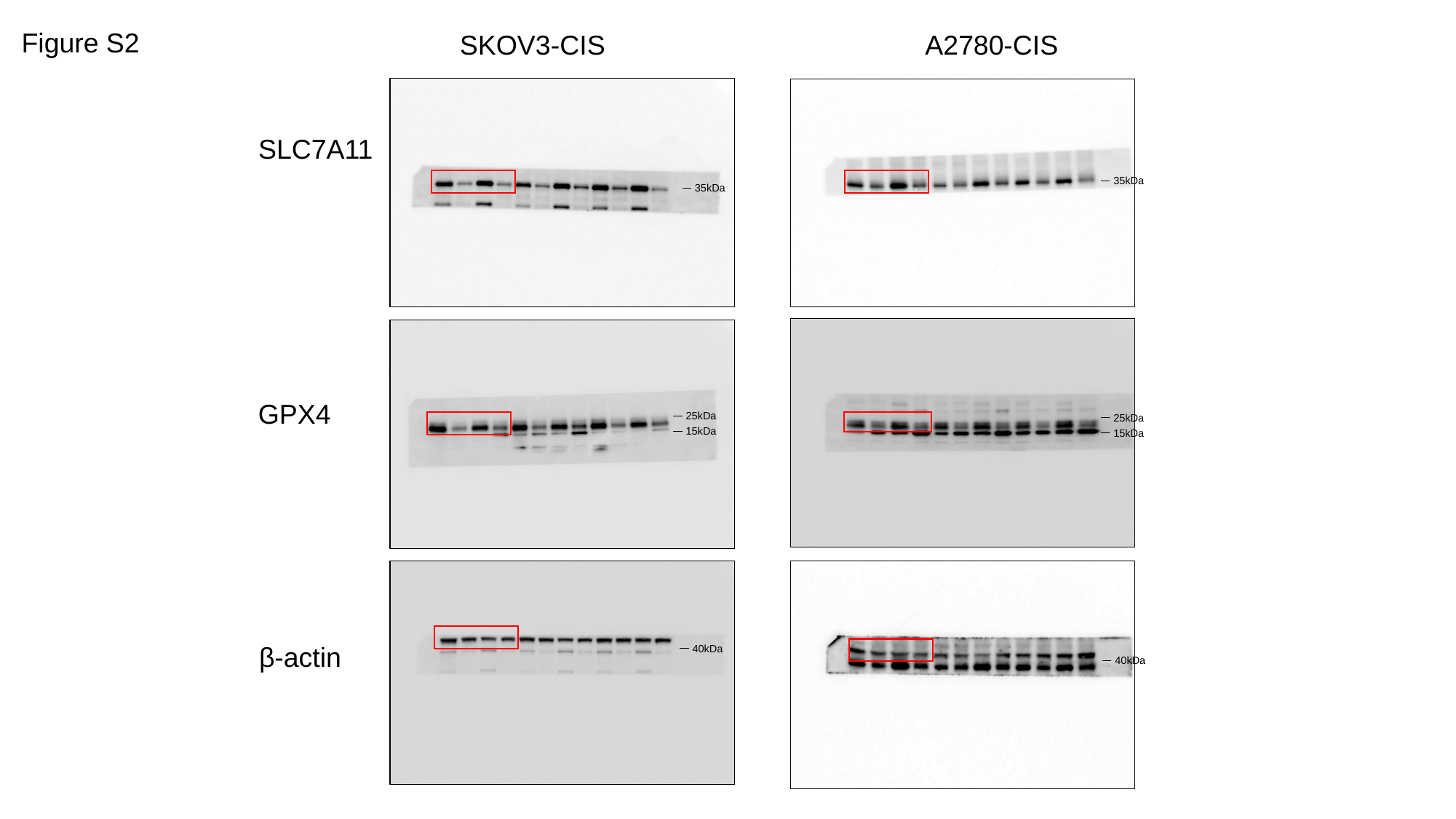

Figure S2
SKOV3-CIS
A2780-CIS
SLC7A11
35kDa
35kDa
GPX4
25kDa
15kDa
25kDa
15kDa
β-actin
40kDa
40kDa

## Slide 6
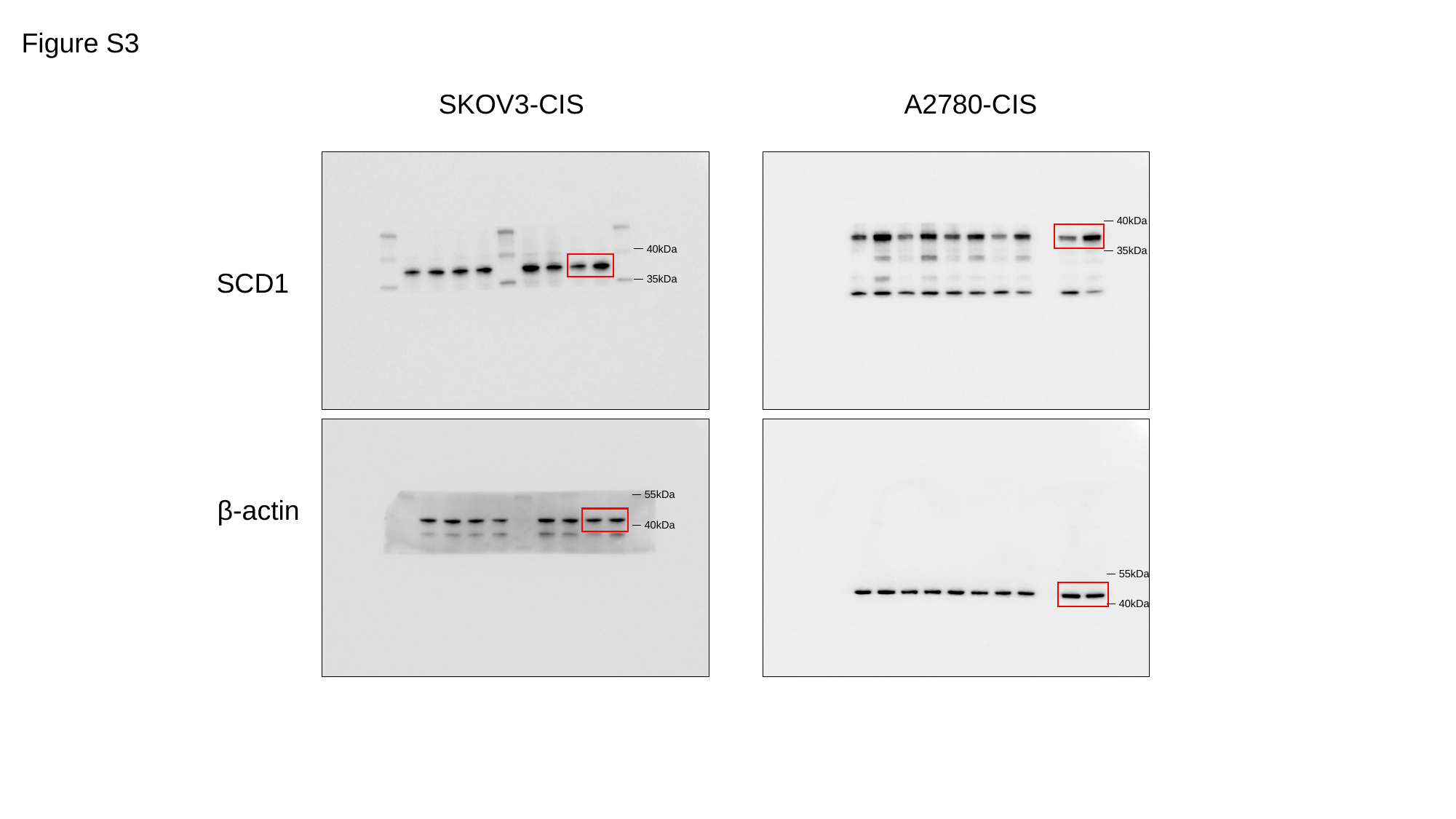

Figure S3
SKOV3-CIS
A2780-CIS
40kDa
35kDa
40kDa
35kDa
SCD1
55kDa
40kDa
β-actin
55kDa
40kDa
