# Supplementary material for: Olaparib synergizes with arsenic trioxide by promoting apoptosis and ferroptosis in platinum-resistant ovarian cancer
Source: Cell Death Dis. 2022 Sep 27;13(9):826. doi: 10.1038/s41419-022-05257-y (PMC9513087; doi:10.1038/s41419-022-05257-y)
Supplement: Supplementary file 2 — Supplementary Materials [file 41419_2022_5257_MOESM2_ESM.docx]

**Supplementary figure legends**

**Olaparib synergizes with arsenic trioxide by promoting apoptosis and ferroptosis in platinum-resistant ovarian cancer**

Sangsang Tang ^1*^, Yuanming Shen ^2*^, Xinyi Wei ^1^, Zhangjin Shen ^1^, Weiguo Lu ^2,3 #^, Junfen Xu ^2#^

**
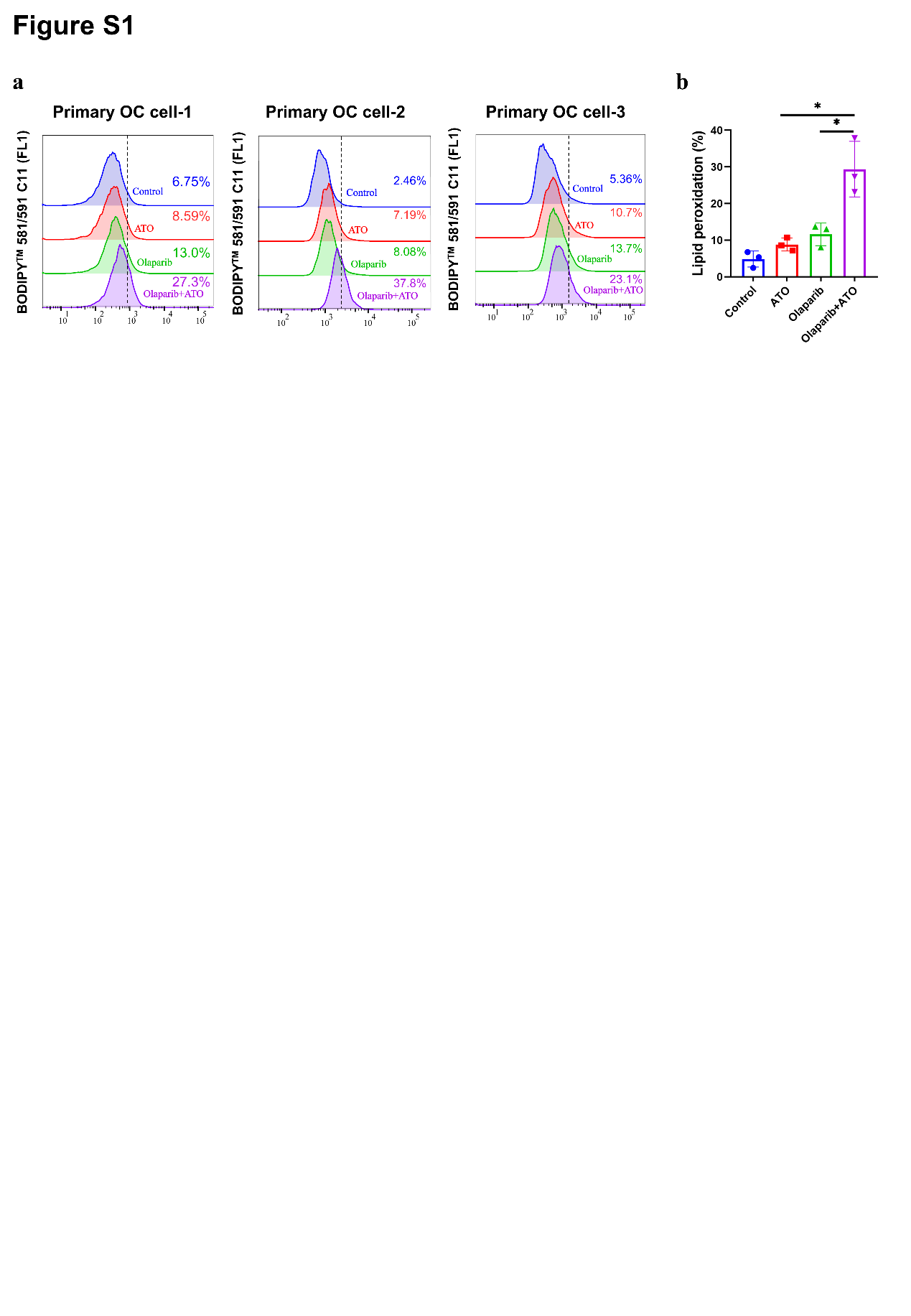
**

**Figure S1.** **Lipid peroxidation of the combined treatment of olaparib and ATO on primary OC cells.** The primary OC cells were treated with olaparib and/ or ATO for 48 h. **(a)** The relative lipid peroxidation levels investigated using BODIPY^TM^ 581/591 C11 fluorescence in three primary OC samples. **(b)** Quantification of lipid peroxidation of three primary OC samples subjected to indicated treatments. Error bars are shown as mean ± SD from 3 primary OC samples.


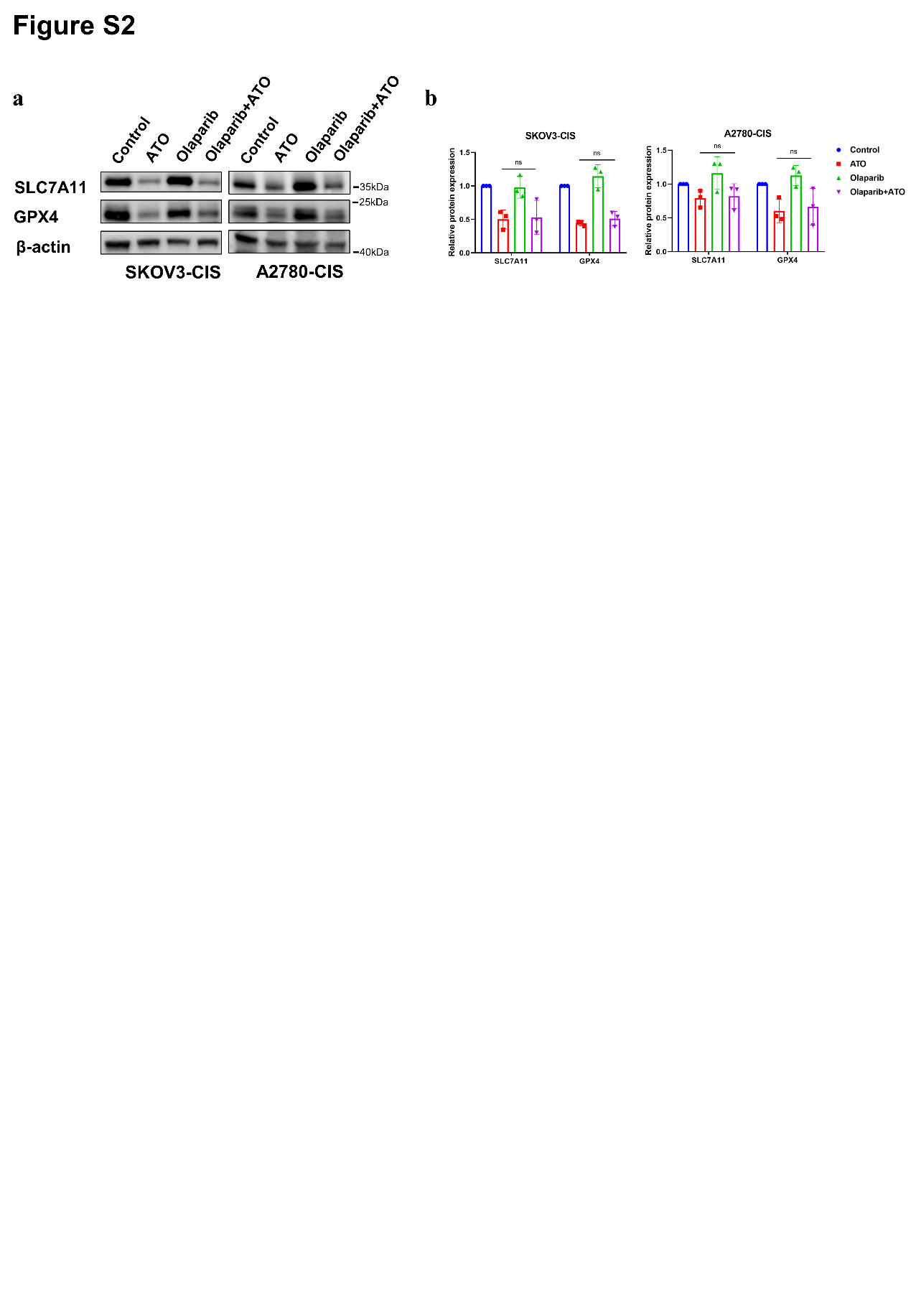


**Figure S2.** Western blot analysis on the expression levels of SLC7A11 and GPX4 in SKOV3-CIS and A2780-CIS cells, detected by Western blot after the indicated treatments. The protein expression levels were normalized with β-actin. The normalized value of the control group was set to 1. Error bars are shown as mean ± SD from 3 independent repeats.


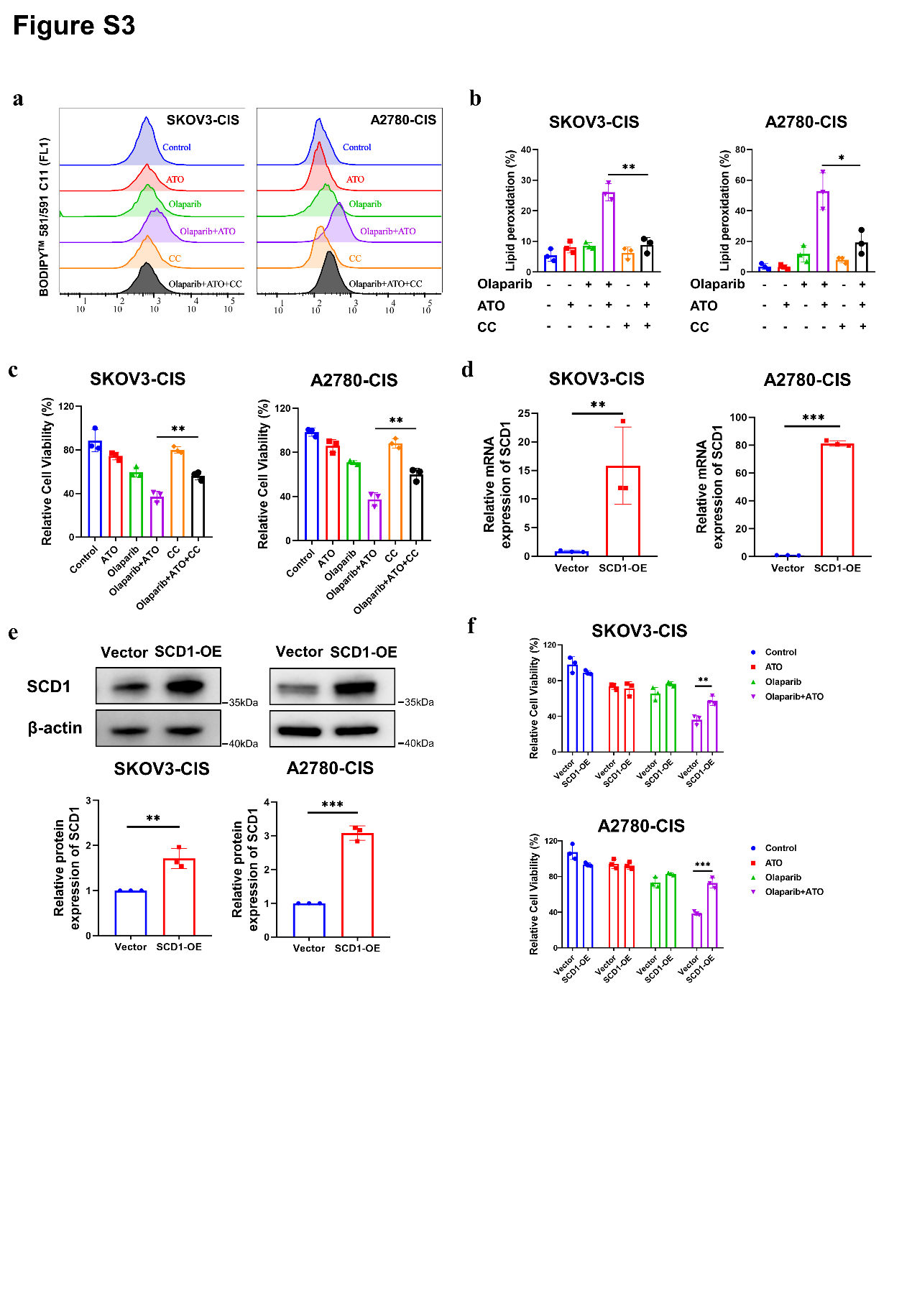


**Figure S3.** Changes in the lipid peroxidation levels **(a and b)** and cell viability **(c)** in SKOV3-CIS and A2780-CIS cells after indicated treatments in the presence or absence of 2μM CC. **(d-e)** SCD1 was overexpressed and determined by qRT-PCR **(d)** and Western blot **(e)**. The relative cell viability of SKOV3-CIS and A2780-CIS cells following indicated treatments where SCD1 was overexpressed or not. Error bars are shown as mean ± SD from 3 independent repeats.
